# Use of Viscous medium to study anthelmintic drug action in *Caenorhabditis elegans*

**DOI:** 10.1038/s41598-024-63090-z

**Published:** 2024-06-04

**Authors:** Jacqueline R. Hellinga, Jürgen Krücken, Hinrich Schulenburg, Georg von Samson-Himmelstjerna

**Affiliations:** 1 Institute für Parasitologie und Tropenveterinärmedizin, Freie Universität Berlin, Robert von Ostertag Str. 7, 14163 Berlin, Germany; 2 Zoologisches Institut, Christian-Albrechts-Universität Zu Kiel, Am Botanischen Garten 1-9, 24118 Kiel, Germany

**Keywords:** *Caenorhabditis elegans*, Anthelmintics, Viscous medium, Larval development, Caenorhabditis elegans, Drug delivery, Animal disease models

## Abstract

*Caenorhabditis elegans* is an appealing tool for experimental evolution and for working with antiparasitic drugs, from understanding the molecular mechanisms of drug action and resistance to uncover new drug targets. We present a new methodology for studying the impact of antiparasitic drugs in *C. elegans*. Viscous medium was initially designed for *C. elegans* maintenance during long-term evolution experiments. Viscous medium provides a less structured environment than the standard nematode growth media agar, yet the bacteria food source remains suspended. Further, the Viscous medium offers the worm population enough support to move freely, mate, and reproduce at a rate comparable to standard agar cultures. Here, the Viscous medium was adapted for use in antiparasitic research. We observed a similar sensitivity of *C. elegans* to anthelmintic drugs as in standard liquid media and statistical difference to the standard agar media through a larval development assay. Using Viscous medium in *C. elegans* studies will considerably improve antiparasitic resistance research, and this medium could be used in studies aimed at understanding long-term multigenerational drug activity.

## Introduction

*Caenorhabditis elegans* occurs in temperate regions of the world, where it commonly inhabits decaying plant matter and feeds on diverse microorganisms^1^. These worms are maintained in the laboratory on nematode growth agar media (NGM) agar and have a typical growth cycle of 3.5 days at 20 °C^2^. Sydney Brenner introduced them into the laboratory to study animal development and their nervous system. Since then, they have become one of the main model systems in biomedical research across diverse fields, including e.g. research on aging, host–pathogen interactions, cell division, cytoskeleton, and drug discovery^3–6^.

*Caenorhabditis elegans* has been used as a model for parasitic worms to understand the mechanism of many anthelmintics and is involved in drug discovery^7–11^. These worms are a valuable model in anthelmintic research as parasitic worms are impossible to cultivate in the laboratory, with the exception of *Parastrongyloides trichosuri*^12^ and lack the same genetic tools as *C. elegans*.

Anthelmintics studied in *C. elegans* include macrocyclic lactones, benzimidazoles, imidazothiazoles, spiroindoles, cyclooctadepsipeptides, and Crystal (Cry) proteins from *Bacillus thuringiensis*^11,13–18^. Macrocyclic lactones interact with ligand-gated ion channels, in particular glutamate-gated chloride channels (GluCls) and in nematodes to a lesser extent to GABA-gated chloride channels^19^. Ivermectin (IVM), a member of the macrocyclic lactones, binds irreversibly to GluCls, which allows an influx of chloride ions, ultimately causing paralysis of the neuronal or muscular membrane^20^. Often, in worms, the pharynx muscles are targeted as well, thus inhibiting the feeding of the worm, and leading to starvation^18^. Therefore, IVM affects both the motility and feeding of the nematode. Moxidectin (MOX) also targets GluCls but has different pharmacokinetics than IVM^19^. The different pharmacokinetics are often considered to be the reason for the higher potency of MOX compared to IVM against resistant isolates of parasitic nematodes in sheep, cattle, horses, and goats^21^. In motility assays using *C. elegans* often slightly higher concentrations of MOX than of IVM are required to achieve half maximal effect change^22^, which can be reversed if worms do not feed, suggesting that both drugs enter the worms via different routes^23^. Benzimidazoles work as anthelmintics by binding to the β-tubulin subunit and preventing microtubule polymerization, inhibiting the microtubule dynamics needed for cells to survive and divide^24,25^. Without the microtubule dynamics within the cell, the worms often become flaccid and paralyzed^25,26^. Lastly, imidazothiazoles are broad-spectrum drugs that act on nicotinic acetylcholine receptors (nAChRs). Of this class, levamisole is the only licensed and most commonly studied drug that targets the body wall muscles and causes spastic paralysis of worms^27^.

Usually, in in vitro assays, *C. elegans* are exposed to the drugs by placing them on nematode growth medium (NGM) agar plates containing the desired concentration of the drug. Nematode growth medium agar was first introduced as a viable media source by Sydney Brenner in 1974^2^. Although this medium is helpful for the easy maintenance of a population and is commonly used for various genetic manipulations such as RNAi and behavioral avoidance experiments, it is limited in effectively characterizing the effects of drugs on a worm population. This is due to the highly dense matrix of the NGM agar, which is maintained to prevent worm burrowing. Organic compounds used as drugs are typically dissolved in DMSO. They often have limited effect on worms on NGM agar due to the little surface area exposed to the drugs at any given time^28,29^. Moreover, the drug has to be added to molten agar (> 55 °C temperature), rapidly mixed and poured into petri dishes. Further NGM agar is a highly complex mixture with undefined compounds of agar and bactopeptone. We speculate that these compounds could decrease the solubility of a drug and lead to it precipitating in NGM agar. By this study design, it is difficult to guarantee a homogenous distribution of the drug in the agar and exclude any temperature dependent drug degradation. Therefore, testing drugs in a liquid-based medium is more desirable to identify their effects on worm populations. One liquid-based medium is liquid NGM which is NGM agar but without agar, it is commonly used for chemical and RNAi screens^8,30^. Another liquid medium, the so-called S medium, is the commonly used liquid medium in *C. elegans* research^31^. It is composed of phosphate buffer with essential additives such as cholesterol, trace metals, magnesium sulfate, and calcium chloride. Unfortunately, S medium does not allow maintenance of worm populations for much more than one generation. This is due to considerable effects on the worm phenotype/morphology such as dauer formation despite the presence of food^31^. Also, worms grown in liquid cultures are often thinner, longer, and often retain their eggs in comparison to worms grown on agar^31,32^. Therefore, any study trying to understand the effects of drugs in an experiment spanning multiple generations such as evolution has limited media sources to use.

Papkou et al.^33^ developed a new Viscous medium (V medium), consisting of S medium supplemented with a nontoxic agent increasing the viscosity of the medium, hydroxypropylmethylcellulose (HPMC), for long-term evolution experiments and high-throughput assays. An initial characterization of V medium with 0.5% methylcellulose revealed that it supports a high efficiency in mating between hermaphrodites and males (indicated by a male ratio between 0.3 and 0.4) and offspring production (e.g., 30–50 offspring per worm after a six day incubation at 16 °C)^8,28, 33^. The previous study used the medium to characterize the coevolutionary dynamics between *C. elegans* as host and bacterial pathogens, demonstrating a more complex model of the Red Queen, consisting of distinct selective processes acting on the two antagonists during rapid and reciprocal coadaptation^33^.

Based on the previous work by Papkou et al. 2019, the objectives of the current study were to develop the V medium further as a basis for dissecting the interaction of the *C. elegans* model with different anthelmintics. In particular, the V medium was tested in larval development assays in comparison to the S medium to determine inferred EC_50_ values and the suitability of V medium for evaluating the effects of anthelmintics.

## Methods

### Worm strains and maintenance and chemicals

*Caenorhabditis elegans* P-glycoprotein 9 knock-out strain tm830 was obtained from the NBRP. Worms were maintained on nematode growth agar media (NGM) agar supplemented with *E. coli* OP50 as a food source at 16 °C using standard manipulation methods (Hope, 1999). Ivermectin (#I8898), moxidectin (#33,746), levamisole (#31,742), and albendazole (A4673) were obtained from Sigma-Aldrich and stored at − 20 °C.

## Viscous medium

V medium has a base of S basal buffer (10 mM Potassium phosphate buffer pH 6.0, 100 mM NaCl). The 450 ml of S basal was heated to 60–70 °C and 4.6 g of (Hydroxypropyl)methylcellulose (H7509, Sigma) was added slowly under constant stirring. The solution was then autoclaved and allowed to cool down. After autoclaving the medium was stirred for 30 min under gentle heat (30–40 °C) until the medium was homogenous. Before use, 10 ml of 1 M potassium citrate pH 6.0, 1.5 ml of 1 M Mg_2_SO_4_, 1.5 ml of 1 M CaCl_2_, and 0.5 ml of 5 mg/ml cholesterol (in 100% ethanol) were added. The media was stored at 4 °C.

### Escherichia coli OP50 preparation

*Escherichia coli *OP50 (*C. elegans* Genetic Stock Center, https://cgc.umn.edu/) was used as a food source for *C. elegans*. Cultures were initiated from a -80 frozen stock and struck out on LB agar (Carl Roth, Germany). Overnight cultures were grown from picking of a single OP50 colony in 500 ml of LB-broth (Carl Roth, Germany) at 37 °C, 200 rpm for 16–20 h. The cells were then pelleted and washed with S basal in 50 ml tubes (4000 rpm, 30 min). *Escherichia coli *pellets (~ 2 g) were resuspended in 20 ml of S basal. The optical density of the *E. coli* was measured at 600 nm using a 1:100 dilution of the final resuspended *E. coli* pellet. The concentrated *E. coli* were stored at 4 °C for 2 weeks until further use.

### Adaptation of *C. elegans* worms to viscous medium

**Figure 1:**
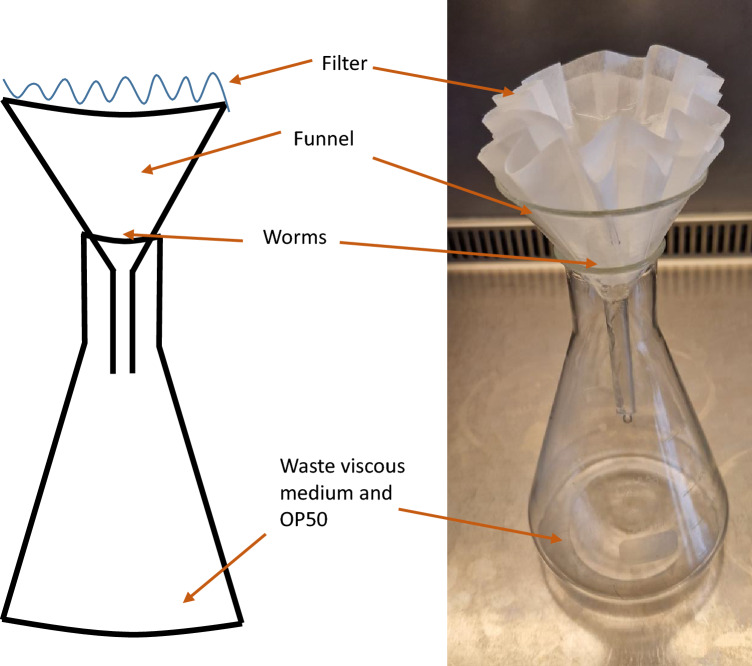
Setup to wash worms on a large scale between generations. Worms were pooled into a 50 ml conical centrifugation tube and centrifuged at 5134 × g for 5 min at 20 °C. The supernatant was removed, and the worm pellet was resuspended. The resuspended worm pellet was washed with approximately 100 ml of S basal in the above system. Worms are placed on a wet filter, and 100 ml of S basal were added periodically. When about 10–15 ml of the solution remained in the filter, a Pasteur pipette was used to remove the worms, and the density of the worms was counted.

*Caenorhabditis elegans* tm830 worms were grown in a 6-well plate in 3.2 ml of V medium supplemented with 10 mM potassium citrate (pH 6.0), 3 mM Mg_2_SO_4_, 3 mM CaCl_2_, 5 µg/ml cholesterol with OP50 as a food source (final optical density OD600 = 1.5). The worms were incubated at 20 °C for 4 days. The wells were washed with 4 ml of S basal to ease the pipetting of the V medium. The worm media mixture was transferred to a 50 ml centrifugation tube and spun at 5134 × g (Eppendorf 5430R) for 5 min at 20 °C. The supernatant was removed, and the worms were placed on top of two layered 150 mm filter papers (Carl Roth Typ600P, #CA20.1) and washed with 100 ml of S basal to remove all remaining V medium (Fig. 1). The worm density was estimated by counting 10 5 µl drops of worms. 3000 worms were placed in each well of a 6-well plate and incubated at 20 °C for 4 days with constant shaking at 150 rpm.

### Synchronization of worms from viscous medium

F2 generation gravid hermaphrodites were washed by adding an equal volume of S basal to each well. The worms were put in a 50 ml centrifugation tube and centrifuged at 5134 × g (Eppendorf 5430R) for 5 min. The supernatant was removed, and the worms were washed as described above and resuspended in 20 ml S basal. A 2 × bleaching solution (1.2% household bleach and 700 mM NaOH) was prepared. The bleaching solution was added at an equal volume to the worm suspension, and it was vortexed on high speed for 6 min, checking for lysis periodically. The bleach/worm suspension was centrifuged for 1.5 min at 5134 × g at 20 °C. The worms were washed three times with 20 ml of S basal and resuspended in 15 ml S basal for incubation overnight at 20 °C with constant shaking at 150 rpm.

### Growth rates in viscous medium

Synchronized *C. elegans* tm830 worms (approximately 1000) at the first larval (L1) stage were added to each well of a 6-well plate containing 3.2 ml of V medium and *E. coli* OP50 as a food source (final OD_600_ = 1.0). Worms were incubated at 20 °C with constant shaking at 150 rpm. Every 24 h, the worms were visualized with a stereomicroscope (Leica MZ 16F) by the removal of 150 µl of media (~ 100 worms) and placed on NGM agar with 10 mM levamisole to paralyze them to image the developing anatomical structures. Four wells were counted at each time point, which was done with 3 biological replicates (n = 12 replicates in total). For each well, the number of all larval stages was counted, and a fraction of the larval stages was determined and averaged. The averages for each biological replicate were graphed with GraphPad Prism 5.0, and the standard deviations and means were determined.

### Worm length and width

Synchronized L1 tm830 worms obtained by bleaching were counted, and approximately 50 worms in quadruplicate were added to 1 ml of S or V medium in a 24 well plate supplemented with *E. coli* OP50 (final OD_600_ = 1). Approximately 60 worms were seeded onto 2 day old NGM agar spotted with *E. coli* OP50. All worms were placed in incubators at 20 °C, with the S and V medium under constant shaking at 150 rpm. After 96 h approximately 30 worms per replicate for each media condition were imaged. Images were collected by ISOCELL HP2 camera and processed using Wormsizer (v 1.2.5). Worm length and mean width were plotted in GraphPad Prism (v 5.0) with mean and standard error shown. *P* values were obtained by one-way ANOVA with post test of Tukey’s multiple comparison test. Not significant (ns) indicates a *P* value above 0.05, * indicates a *P* value below 0.05, and *** indicates a *P* value lower than 0.0001.

### Larval development assay

For assays with S basal, synchronized L1 tm830 worms obtained by bleaching were counted, and approximately 100 worms were added in 24 µl S basal to every well of a 48-well microtiter plate already containing 200 µl S medium, 25 µl *E. coli* OP50 (OD_600_ = 2), and 1 µl anthelmintic was added. For all anthelmintics, serial dilutions were prepared in 100% DMSO or S basal (levamisole). For ivermectin, the final concentration range was 0.07–8 nM with a 1.4 fold dilution between concentrations, 0.02–6 nM with a 1.5 fold dilution for moxidectin, 0.9–100 µM with a 1.4 fold dilution for levamisole, and 2–600 µM with a 1.5 fold dilution for albendazole. Drug concentrations were done in triplicate, and four biological replicates were done on different days for each drug/medium combination. Worms were incubated at 20 °C with constant shaking at 150 rpm. After a 48–55 h incubation, the development was stopped by adding Lugol's iodine. Worms that developed into either the L4 or adult stage were counted as fully developed, while L1, L2, and L3 were considered not developed. The EC_50_ values were determined by performing the variable slope log_10_(inhibitor concentration) vs. percent development response curve as logistic regression (Variable slope (four parameters)) analysis using GraphPad Prism 5.0. EC_50_ values were compared using the extra sum of squares F-test within GraphPad Prism. *P* values < 0.05 were considered to be statistically significant.

Experiments with V medium were performed as described for S basal, but the *C. elegans* tm830 worms synchronized for the larval development assay had been grown in V medium in a 6-well plate with 3000 worms per well for 2 generations prior to the experiment. Each generation was transferred as described above. Every well of a 48-well microtiter plate had 200 µl V medium.

Assessment of development variability was done by graphing the percentage of developed worms in DMSO controls for ABZ, IVM and MOX from S and V medium larval development assays in GraphPad Prism 5.0. Bartlett’s test was performed in R studio (v 4.2.0).

## Results

### Working with viscous medium

The adaption of the V medium from a long-term evolution experiment^33^ to a short-term phenotypic assay included changing media supplements of the V medium (Table 1). Papkou et al. found that adding trace metal solutions to V medium did not affect the worms' growth. We tested this idea and found that while no short-term effect was found on the worms grown in V medium with trace metal solutions over time, it was observed that there was a delay in the growth rate. Therefore, the trace metal solution was removed from further V medium experiments due to concerns that it was causing a change in the worms that we could not quantify.

Further, the optimum OP50 density for worm populations ranging from 500 to 3000 worms had to be found in adapting the V medium for use with anthelmintics. Too much OP50 hampered viewing of the worms during counting stages, making it impossible to see if any worms survived treatment. Also, increasing the worm population within a well required an increased amount of OP50. We found that any worm population of 500 or less would not starve with an OP50 optical density (OD_600_) of 0.5 in the well. A 1000-worm population needed an OP50 density of 1 in the well to prevent starvation, and the 3000-worm population required an OP50 density of 3 in the well.

**Table 1:** Composition of Viscous medium.

Component^a^	Viscous medium from Papkou et al. 2019	Viscous Medium from this study
S basal Base: 10 mM Potassium Phosphate Buffer pH 6.0, 100 mM NaCl	✔	✔
0.8% Hydroxypropylmethylcellulose	✔	✔
10 mM Potassium Citrate buffer pH 6.0	✔	✔
Trace Metal Solution (55 uM disodium EDTA, 24 uM FeSO_4_ ·7H_2_O, 10 uM MnCl_2_·4H_2_O, 10 uM ZnSO_4_·7H_2_O, 1 uM CuSO_4_·5H_2_O)	✔	
3 mM Mg_2_SO_4_	✔	✔
3 mM CaCl_2_	✔	✔
5 µg/ml cholesterol	✔	✔

^a^Final concentration in Viscous medium.

### Life cycle of mutant Δ*pgp-9* (tm830) in V medium

**Figure 2:**
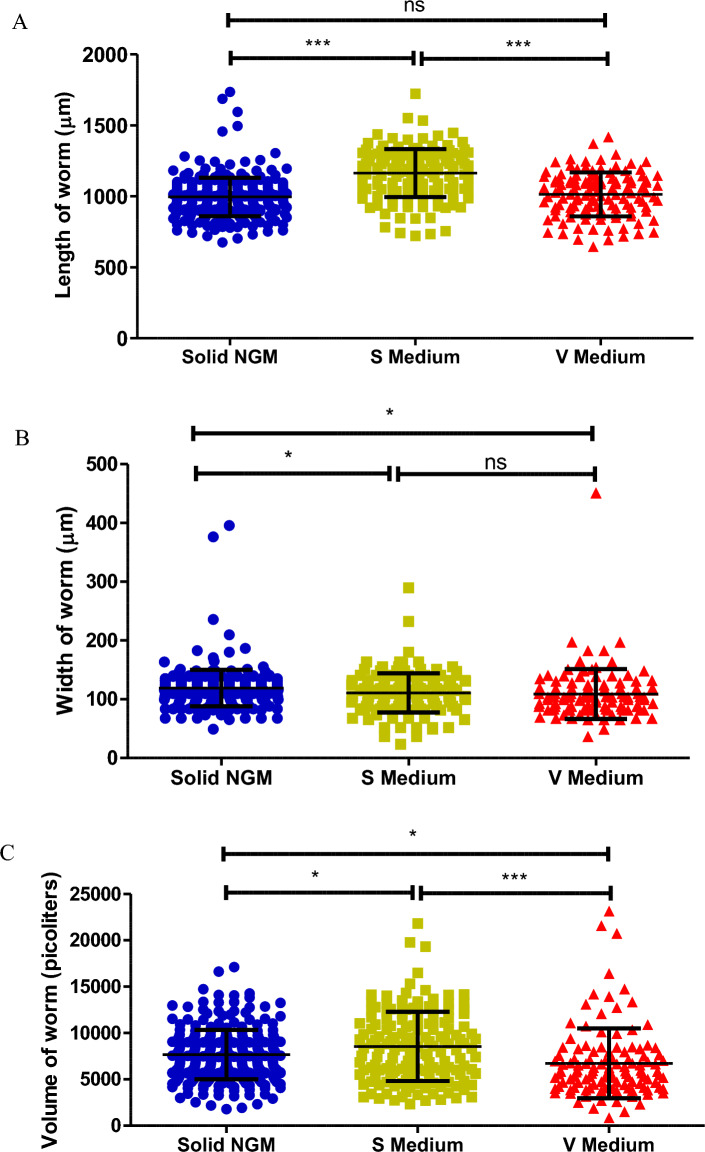
*Caenorhabditis elegans* tm830 lengths (**A**) and mean width (**B**) after 72 h incubation of L1 in either S or V medium or on NGM agar. Length, width, and volume were obtained by Wormsizer (v 1.2.5). Graphs show individual values for approximately 100 worms for each condition, means and SD are shown. P value obtained by 1way ANOVA with post test Tukey's multiple comparison test. ns indicates P value above 0.05, * indicates a P value below 0.05, and *** indicates a P value lower than 0.0001.

We then tested how long a generation would take to develop, starting at an L1 stage. The experiment started with 1000 L1s per well in a 6-well microtiter plate. In an initial experiment, worms were incubated at 16 °C and 20 °C without shaking of the microtiter plate, resulting in death of all worms within 24 h, presumably due to a lack of oxygen. Therefore, all further experiments involved shaking the worms in the V medium at a constant speed of 150 rpm at 20 °C. The worms were analyzed for developmental stages every 24 h after the initial time point. Approximately 100 worms were counted in three different wells per time point. After 24 h, the majority of the worms were still in the L1 stage with a fraction of 0.896, with some having molted into the L2 stage (0.104) (Table 2). After 48 h, the worms were mainly at the L2 stage (0.646), with some L3 worms in the population (0.142) (Table 2). At 72 h, the worms were at the L3 stage (0.607), with a few L4/adults (0.125) present. The L4 worms were identified by the characteristic vulva bubble. At 96 h, most worms were L4/adults (0.644), with a few L1 worms (0.260) (Table 2). These L1 worms represented the offspring from the adults seen the days before (Fig. 2). With this growth cycle, it was concluded that the average time for molting and development is similar to the growth cycle observed on nematode growth agar^31^.

**Table 2:** The fraction of worms with standard deviation in the various larval stages, as counted every 24 h over 4 days.

Hours	L1 stage	L2 stage	L3 stage	L4/adult stage
24	0.896 ± 0.032	0.104 ± 0.032	0	0
48	0.212 ± 0.033	0.646 ± 0.071	0.142 ± 0.051	0
72	0	0.267 ± 0.036	0.607 ± 0.030	0.125 ± 0.024
96	0.260 ± 0.064	0.017 ± 0.031	0.075 ± 0.022	0.644 ± 0.081

Additionally, we measured the average length, width, and volume of the *C. elegans* tm830 worms grown in either S medium, V medium or NGM agar at 20 °C for 72 h. The worms grown on NGM agar had an average length of 995.6 µm (± 135.7 µm, S.D.) and width of 119.1 µm (± 31.21 µm, S.D) (Fig. 2). Worms that were grown in S medium had an average length of 1164 µm (± 169.1 µm, S.D.) and width of 110.9 µm (± 33.27 µm, S.D.) (Fig. 2A,B). The difference of length and width between worms grown on NGM agar and S medium was statistically significant (Tukey’s method, *P* value < 0.0001) and this is similar to what has already been previously reported in the literature^34^. However, there was a small significant difference (Tukey’s method, *P* value < 0.05) in the volume of the worm although it has reported that though worms grown in S medium are longer and thinner than worms grown on NGM agar their volume should be similar (Fig. 2C)^35^. Worms grown in V medium had an average length of 1014 µm (± 155.0 µm, S.D.) (Fig. 2A). There was no significant difference in length between worms grown on NGM agar and those grown in V medium (ANOVA, *P* value 0.4938). However, there was a significant difference between S medium and V medium (Tukey’s method, *P* value < 0.0001). In addition, worms grown in V medium had the smallest width of 108.8 µm (± 42.38 µm, S.D.). The difference in width was not statistically different to S medium (ANOVA, P value 0.6391) but different to NGM agar (Tukey’s method, *P* value < 0.05) (Fig. 2B). Lastly, the volume of the worms grown in V medium was statistically different to both S medium (Tukey’s method, *P* value < 0.05) and NGM agar (Tukey’s method, *P* value < 0.05) (Fig. 2C). Therefore, worms grown in V medium have less volume than worms grown in S medium or on NGM agar.

### Response to anthelmintics

Larval development assays are a standard assay to determine a drug’s effectiveness and it has the advantage that it can be used for all anthelmintic drug classes^36,37^. We tested the impact of the V medium on the EC_50_ value of *C.* *elegans* tm830 worms when exposed to various classes of anthelmintics. The EC_50_ values from the V medium were compared against EC_50_ values obtained from larval development assays of the worms in the standard S medium and NGM agar.

**Figure 3:**
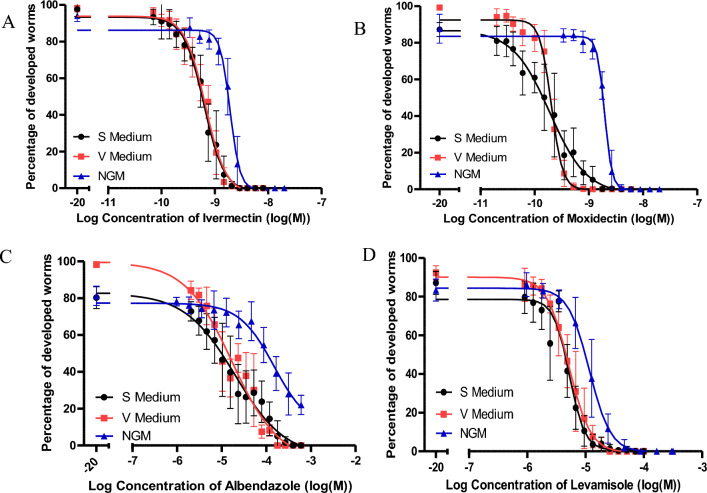
Concentration–response-curves of *C. elegans* tm830 worms in either S or V medium when exposed to (**A**) ivermectin, (**B**) moxidectin, (**C**) albendazole, and (**D**) levamisole. Worms were exposed to drug concentrations in triplicate and four independent biological replicates were included. Worms that developed into either the L4 or adult stage were counted as fully developed, while L1, L2, and L3 were considered not developed.

The first class of drugs tested were macrocyclic lactones which target GluCls, which leads to a flaccid response in the nematodes. Exposure of worms to IVM led to similar EC_50_ values with no significant difference between V (0.68 nM) or S medium (0.63 nM) (Fig. 3A, Table 3). Interestingly, there was a significant difference (*P* value < 0.0001) for the EC_50_ value when the worms were exposed to IVM in either S or V medium in comparison to NGM agar. On NGM agar the EC_50_ value was 1.92 nM, which was a 3.1 fold change in comparison to S medium and a 2.8 fold change in comparison to V medium (Table 3).

**Table 3:** EC_50_ values of  *C. elegans* tm830 worms to various anthelmintics determined by larval development assays.

Drug	Medium	EC_50_ ± SE [nM]	95% CI [nM]	R^2^	Fold change	*P* Value
Ivermectin	S	0.63 ± 0.01	0.57–0.67	0.952	0.93^a^	0.236^b^
	V	0.68 ± 0.01	0.61–0.70	0.967		
	NGM Agar	1.92 ± 0.05	1.82–2.01	0.965	3.05^c^	< 0.0001^e^
					2.82^d^	< 0.0001^f^
Moxidectin	S	0.18 ± 0.04	0.15–0.22	0.910	0.90^a^	0.407^b^
	V	0.20 ± 0.009	0.19–0.21	0.977		
	NGM Agar	1.94 ± 0.006	1.89–2.01	0.981	10.77^c^	< 0.0001^e^
					9.70^d^	< 0.0001^f^
Levamisole	S	5250 ± 18	4830–5707	0.919	1.02^a^	0.633^b^
	V	5100 ± 17	4703–5531	0.949		
	NGM Agar	11,590 ± 21	10,500–12,780	0.961	2.21^c^	< 0.0001^e^
					2.27^d^	< 0.0001^f^
Albendazole	S	19,980 ± 91	13,396–34,434	0.840	1.16^a^	0.546^b^
	V	17,140 ± 58	13,152–22,335	0.911		
	NGM Agar	150,800 ± 141	78,740–288,700	0.861	7.54^c^	< 0.0001^e^
					8.79^d^	< 0.0001^f^

Worms that developed into either the L4 or adult stage were counted as fully developed, while L1, L2, and L3 were considered not developed.

^a^Calculated as EC_50_ S medium/EC_50_ V medium.

^b^Sum of squares F test comparing S medium to V medium.

^c^Calculated as EC_50_ NGM agar/EC_50_ S medium.

^d^Calculated as EC_50_ NGM agar/EC_50_ V medium.

^e^Sum of squares F test comparing of NGM agar to S medium.

^f^Sum of squares F test comparing of NGM agar to V medium.

Exposure to MOX, another macrocyclic lactone, also produced similar EC_50_ values for both V (0.20 nM) and S medium (0.19 nM) (Fig. 3B, Table 3) and the difference was not significant (*P* = 0.407). As above, the results for S or V medium were significantly different to those on NGM agar. This was due to the EC_50_ value of 1.94 nM on NGM agar, which is 10.77 and 9.70 fold higher than those for S and V medium, respectively (Table 3).

Albendazole, an anthelmintic belonging to the benzimidazole class, was also tested (Sharp, 1997). In the S medium, the EC_50_ value was 19.98 µM which was slightly higher than the EC_50_ value of 17.14 µM for the V medium (Fig. 3C, Table 3) but again this difference was not significant (*P* value = 0.546). However, the EC_50_ value in NGM agar was 150.8 µM, which is 7.54 fold higher than the EC_50_ value found in S medium and 8.79 fold higher than in V medium. The difference in the EC_50_ value between NGM agar and S/V medium was statistically significant (*P* value < 0.0001). The development curve for NGM agar was not able to reach a 0% development stage due to lack of solubility of albendazole in DMSO for the stock solution and for the serial dilution in agar. More DMSO could not be added to the wells as DMSO concentrations higher than 1% would inhibit worm development^38^.

Lastly, exposure of worms to a cholinergic agonist, levamisole, led to similar EC_50_ value of 5.25 µM in S and 5.10 µM in V medium, respectively (Fig. 3D, Table 3) indicating no significant difference (P = 0.633). The EC_50_ value on NGM agar was again significantly higher with 11.59 μM (Fig. 3D), which is twofold higher than the EC_50_ value obtained for S or V medium (*P* value < 0.0001) (Table 3).

**Figure 4:**
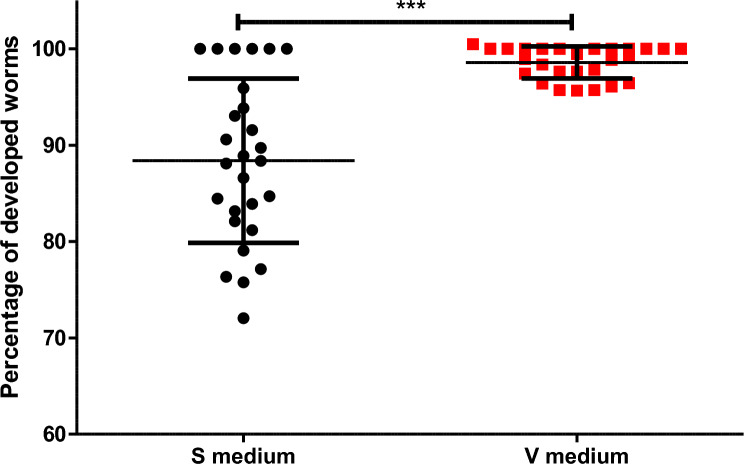
Development of *C. elegans* tm830 worms in S and V medium. Worms were measured for development after a 48 h incubation at 20 °C. Mean and standard deviation are reported. *** indicates a *P* value < 0.0001.

Overall, our study demonstrated that more worms developed successfully in the V medium than in the S medium (Fig. 4). In V medium, the development percentage was 98.58% ± 1.65 whereas the S medium had a development percentage of 88.38% ± 8.52 (Table 4). Further, the variation in developmental rate is much higher in S medium than in V medium (Bartlett’s test, *P* value = 1.505 × 10^–12^) (Table 4). Therefore, the V medium was more consistent in obtaining developed worms than the S medium.

**Table 4:** Development of tm830 worms in either S or V medium for 48 h incubation at 20 °C.

Medium	S	V
Mean^a^	88.38 ± 8.52	98.58 ± 1.65
P value^b^	< 0.0001	
P value^c^	1.505 e-12	

^a^Standard deviation is reported along with mean.

^b^*P* value determined by unpaired t test.

^c^*P* value determined by Barlett’s Test.

## Discussion

The V medium was previously shown to be highly effective for performing evolution experiments with coevolving *C. elegans* and pathogenic *Bacillus thuringiensis*^33^. In this current study, the V medium was redesigned to test whether it can be used to determine effects of anthelmintic drugs as an alternative or even replacement to the commonly used S medium for phenotypic assays. More importantly, the use of V medium with anthelmintics implies that long term experiments could be designed to explore how anthelmintic resistance is generated. Since anthelmintic resistant worms were generated in the past on NGM agar (James and Davey, Menez 2016)^21,39^ the use of V medium may permit a more comprehensive, large-scale evaluation of resistance evolution, as the V medium facilitates parallel processing of a larger number of nematode populations^33^. Moreover, as seen with a higher EC_50_ generated on NGM agar the use of V medium could potentially lead to worm populations with a more realistic EC_50_ value as the worms are surrounded by the drug and not interacting with it through their cuticle contacting the agar surface. Also, as observed in Miltsch et al. the use of NGM agar for a phenotypic thrashing assay resulted in incomparable data, in contrast to what was observed before in any phenotypic assay using S medium^40^. The authors attributed this change of the data due to a long time delay between experiments but it could be caused by the crudely defined components of agar.

In our current study, *C. elegans* worms responded to anthelmintics at a similar EC_50_ value in V as in S medium. Moreover, we found that the V medium was easier to work with and manipulate. In our experience, it was however essential to adapt the worms to V medium for the success of any subsequent phenotypic assays. The adaptation over one to two generations in V medium from NGM agar allows any physiological and molecular changes associated with the new medium to occur before the planned experiments. A previous report regarding the change of the transcriptome of *C. elegans* N2 worms upon transfer from a solid to a liquid medium revealed that there was downregulation of genes for cephalic sheath cells and upregulation of genes in the TGF-beta signaling and WNT signaling pathways (Celen et al. 2018)^41^. However, use of V medium to support multiple generations from the initial thawing to phenotypic assays should prevent any sudden medium-dependent physiological responses by the worms, which otherwise may then interfere with the response to the anthelmintic drug.

The overall health of the worms was similar in V medium as on NGM agar. The growth of the worms in V medium was similar to the growth cycle of worms on NGM agar^34,35^. Other liquid sources used to grow worms such as the axenic liquid media (*C. elegans* maintenance medium) commonly used in metabolism studies can lead to delayed and often highly distorted development^42^. Therefore, V medium provides support to the worms to maintain their growth cycle. Further, the average length of worms grown in V medium was similar to those grown on NGM agar. This could be due to the stability of the cellulose matrix within V medium. Worms grown in S medium were statistically longer than worms grown on NGM agar or V medium. Worms grown in V medium were statistically smaller in width than those grown on either NGM agar or S medium and they had a lower volume overall. This indicates that though the worms grown in V medium have the same length as those grown on NGM agar they overall are smaller than worms grown in S medium or on NGM agar. However, V medium does provide an environment that allows *C. elegans* to fully develop into adult worms in a completely defined medium and without growing on (NGM) agar.

Notably, our results show that when using the V medium to test various anthelmintics there were no significant differences in EC_50_ values compared to the S medium. The highest difference between both media was observed for albendazole and even here only a 16% higher EC_50_ value was obtained for S medium. In contrast, when comparing the EC_50_ between NGM agar and any of the liquid media, it was apparent that the EC_50_ value changed substantially and significantly, up to more than 280% for IVM. This could be because anthelmintics did not diffuse evenly in the NGM agar, or the worms were not immersed in medium containing the drugs. Therefore, the drug was not able to interact with the full worm cuticle but only a part of it exposed to the agar. Therefore, these results suggest that either S or V medium should be used when conducting larval development assays with anthelmintics, whereas assays on NGM agar appear to produce high EC_50_ values. Lastly, worms grown in V medium had a more even rate of development than in S medium. Moreover, worms in S medium were 10% less developed than in V medium and more variable. This variability was determined to be significant between the two medias by Bartlett’s test with a p value of 1.505 × 10^–12^ (Table 4). The difference in development can also be seen in the development assays as the V medium curve is higher than the S medium curve for all of the anthelmintics tested.

Overall, we can conclude that V medium appears to be a medium type that would support long-term multigenerational drug activity studies. It can support worm growth at a similar rate as is observed on NGM agar, since worms develop as they do on NGM agar. Moreover, the previous study by Papkuo et al. 2019 already showed that the V medium supports efficient mating of nematodes, in contrast to the liquid S medium^33^. At the same time, when worms are exposed to anthelmintics their response is similar to that in S medium. The cellulose matrix in the V medium is dense enough to support worm growth yet it allows the worms to be fully interactive with anthelmintics as in liquid media. Therefore, the V medium could be used for long-term multigenerational drug activity studies.

The use of the V medium could also facilitate analysis of other drug delivery systems since *C. elegans* have been used in high throughput drug screens to understand drug mechanics, neurodegenerative diseases and test antimicrobials and antifungals^43–46^. We propose that instead of using S medium for many of these assays that only span one generation to use instead V medium to build multigenerational experiments. This would allow researchers a larger downstream prospective on the effects of these drugs especially on any phenotypic defect that could affect offspring viability and performance. As the V medium was previously shown by Papkou et al. (2019) to be effective in evolution experiments, it would be interesting to study other pathogens such as *Pseudomonas* or *Stapholycoccus* and see if they have the same mechanism of killing as observed before on agar^6^. Further, V medium allows us to study the effects of pathogenic bacteria and also other types of drugs, such as antimicrobials or additional anthelmintics, on the progeny of the worms as well as resulting resistance evolution, which is not possible in the standard S medium.

## Data Availability

All data generated or analysed during this study are included in this published article.
